# Seasonal variation in the international normalized ratio of neonates and its relationship with ambient temperature

**DOI:** 10.1186/s12887-016-0639-1

**Published:** 2016-07-19

**Authors:** Shigeo Iijima, Katsuyuki Sekii, Toru Baba, Daizo Ueno, Akira Ohishi

**Affiliations:** Department of Regional Neonatal-Perinatal Medicine, Hamamatsu University School of Medicine, Hamamatsu, Japan

**Keywords:** International normalized ratio, Coagulometer, Seasonal variation, Temperature, Humidity, Neonate

## Abstract

**Background:**

The morbidity and mortality rates due to cardiovascular events such as myocardial infarction are known to exhibit seasonal variations. Moreover, changes in the ambient temperature are reportedly associated with an increase in these events, which may potentially involve blood coagulation markers. Bleeding due to vitamin K deficiency in neonates, which is associated with high mortality and a high frequency of neurological sequelae, is more commonly observed during the summer season and in warm regions in Japan. To determine the presence of seasonal variation and the influence of ambient temperature on blood coagulation markers in healthy term neonates, we assessed the international normalized ratio (INR) values measured using CoaguChek XS.

**Methods:**

We studied 488 consecutive healthy term neonates who were born at a perinatal center between July 2012 and June 2013. The INR values were measured using CoaguChek XS in 4-day-old neonates who received nursing care in the newborn nursery throughout the duration of hospitalization. The seasonal variations in the INR values and environmental effects on the INR were assessed.

**Results:**

The mean monthly INR values peaked in July (1.13 ± 0.08), whereas the lowest values were observed in January (1.05 ± 0.08). Higher levels of INR were observed during the summer season (June to August) than during the winter season (December to February). Simple linear regression analysis indicated the presence of weakly positive but significant correlations between INR and outdoor temperature (*r* = 0.25, *p* < 0.001), outdoor relative humidity (*r* = 0.19, *p* < 0.001), and room relative humidity (*r* = 0.24, *p* < 0.001), and the presence of a significant negative correlation between INR and room temperature (*r* = −0.13, *p* = 0.02). Furthermore, multiple linear regression analysis showed that only outdoor temperature significantly influenced the INR.

**Conclusions:**

A seasonal variation in the INR values was observed among neonates, possibly due to the variation in ambient temperature. Even though the neonates received nursing care in the newborn nursery that was constantly air-conditioned, the outdoor temperature was the most influential factor on INR.

## Background

Blood coagulation is known to exhibit seasonal variations [1, 2]. Moreover, these changes were also found to be associated with seasonal variations in the rate of myocardial infarction, deep vein thrombosis, and pulmonary embolism, which occur more frequently during the colder months of the year [3–5]. Several studies have shown that changes in ambient temperature are associated with higher morbidity and mortality due to cardiovascular events [6, 7]. In fact, coagulation abnormalities are common and represent a clinically important problem in the neonatal population. Bleeding due to vitamin K deficiency (VKDB) is a serious condition that is associated with high mortality and a high frequency of neurological sequelae in surviving children [8]. A previous nationwide survey in Japan demonstrated that the idiopathic type of VKDB more frequently develops during the summer season and in the southern part of Japan [9]. Based on these findings, we hypothesized that the coagulation profiles of neonates may exhibit a seasonal variation, possibly due to temperature variations. In the adult population, several circulating hemostatic biomarkers have been found to show seasonal fluctuations, which could be related to the ambient temperature [10–14]. However, the coagulation systems of neonates significantly differ from those in adults, and to the best of our knowledge, no study has assessed the seasonal variations and effects of temperature on neonatal coagulation. We previously described the reference interval for the international normalized ratio (INR) by using a portable coagulometer, CoaguChek XS (Roche-Diagnostics, Mannheim, Germany), among healthy term neonates [15]. In the present study, we conducted post-hoc analyses of these INR data to determine the presence of seasonal variation and the influence of ambient temperature and humidity on blood coagulation in neonates.

## Methods

### Subjects and data collection

The data of subjects in this study are identical to those used in our previous study published in this journal [15]. The data were collected from consecutive healthy neonates born at Hamamatsu University Hospital from July 1, 2012, to June 30, 2013. The neonates were born at full term (37–41 weeks of gestation) with a normal birth weight (2500–3999 g); 2 mg of vitamin K syrup (Menatetrenone Kaytwo Syrup, Eisai Co., Ltd., Tokyo, Japan) was orally administered 6–12 h after birth. The neonates who were hospitalized in the neonatal intensive care unit (NICU) and whose mothers had known coagulation disorders and/or took medications that could affect neonatal coagulation system were excluded from this phase of the study. After obtaining informed consent from the parents, the neonates were prospectively enrolled in the present study.

The mothers of the neonates were discharged from the maternity ward in 4 or 5 days after delivery, and both a medical examination and an inherited metabolic disorder screening (mass screening) were performed before discharge on day 4 after birth. During the neonatal health checkup, a capillary whole blood sample was obtained via a heel prick at the time of blood sampling for mass screening. The coagulation screening test comprised prothrombin time (PT) using a portable coagulometer, CoaguChek XS, as well as INR. The first drop of capillary blood (at least 8 μL) obtained from a single heel prick was applied to the test strip, which was already inserted into the CoaguChek XS device. Thereafter, a whole blood sample (approximately 40 μL) was collected into a heparinized capillary tube for the measurement of hematocrit values on a routine basis. In addition, another whole blood sample (maximum of 200 μL) was collected on the filter paper for mass screening. Blood samples were drawn by 4 dedicated and experienced neonatologists who had received training in the use of the CoaguChek XS. The hematocrit was measured by using the microhematocrit method.

Every neonate in our hospital received care in the newborn nursery, which had no open doors or windows and was air-conditioned at approximately 26 °C throughout the day, until the mother was discharged from the maternity ward. No neonate was discharged before the blood sampling, and he or she was not exposed to outdoor environmental factors. All the evaluations began at 10:00 h, and were performed in the newborn nursery.

### Portable coagulometer

CoaguChek XS is a small, battery-powered, handheld meter that is portable and efficient. It measures the INR using whole blood obtained by capillary puncture. The procedure involves the insertion of a test strip into the monitor and the application of a drop of blood (8 μL) onto the test strip. The monitor uses an electrochemical method to determine the PT, via the activation of coagulation with recombinant human thromboplastin within the test strip. The mean international sensitivity index (ISI) for the CoaguChek XS PT test is 1.01. The PT is then converted to the INR by using the ISI that was previously recorded and encoded onto the chip for each lot of test strips. The INR value is usually obtained in <1 min. The CoaguChek XS system has quality control functions integrated into the meter and test strips. Therefore, the meter automatically runs its own quality control test as part of every blood test without an examiner running quality control tests using liquid quality controls. We have previously demonstrated that the CoaguChek XS device is safe, fast, and convenient for performing INR assays in neonates. Moreover, we confirmed the reference interval for INR among 4-day-old neonates by using the CoaguChek XS, wherein the median value was 1.10 and the range was 0.90–1.30 [15]. The present study was supported by the intramural research grant program of Hamamatsu University School of Medicine, which was used to cover the CoaguChek XS machine and disposable test strips.

### Seasons

Standard dates were used to define each season during the study period. Summer was considered to range from June 1 to August 31, autumn was considered to range from September 1 to November 30, winter was considered to range from December 1 to February 28, and spring was considered to range from March 1 to May 31.

### Collection of data of climatic conditions

Data of the climatic variables on the day on which blood samples were taken were obtained from the website of the Japan Meteorological Agency; the data included readings of maximum, minimum, and average temperatures (°C), as well as the average relative humidity (%). These were measured at the Hamamatsu Local Meteorological Observatory, which is located approximately 1 mile from our hospital. Japan is a chain of long islands running from north to south, and Hamamatsu is a city located in the central region with a mild climate.

### Collection of data of thermal environment in the newborn nursery

The room temperature and relative humidity were continuously monitored by an indoor digital thermohygrometer (SATO KEIRYOKI MFG. CO. LTD, Tokyo, Japan); the values were recorded at a similar time in the morning on a daily basis. This device is reported by the manufacturer to be accurate to within ±1 °C for temperature and ±5 % for relative humidity. The digital thermohygrometer was attached to a wall surface in the newborn nursery and was located at the altitude of 1.2 m from the floor. The place at which blood sampling was performed was approximately 1.5 m away from the location of the thermohygrometer.

### Statistical analysis

The results are expressed as mean (± standard deviation [SD]). Categorical variables are reported as counts and percentages. The correlation between variables was evaluated using Pearson’s correlation coefficient. The one-way ANOVA was used to evaluate variables in the 4 different seasons. Simple and multivariate regression analyses were used to evaluate the influence of outdoor/room temperature and relative humidity on the INR. We think that these analyses are reliable because the residuals were normally distributed while the INR values were not normally distributed when tested by the Shapiro-Wilk test in our previous study [15]. As for identifying outliers, none were detected by the Dixon’s method in the previous study [15]. In the present study, we reviewed outliers using a different method (a boxplot method), and the method showed 5 outliers. However, as a result of having examined these outliers closely, there was no factor that could explain the necessity of excluding them. Therefore, we proceeded with statistical analyses without excluding the outliers. The Statistical Package for Social Sciences (SPSS version 18, Tokyo, Japan) for Windows was used to manage and analyze the data. A p value of <0.05 was considered to be statistically significant.

## Results

**Fig. 1:**
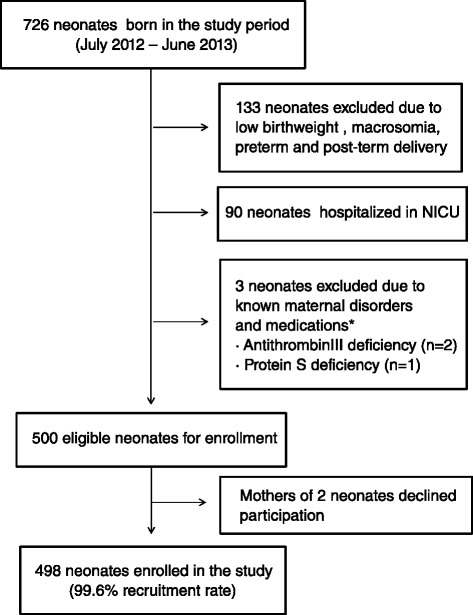
Flow chart of subject recruitment. *, Maternal disorders or medications that affect neonatal coagulation system

The subject recruitment flow chart is shown in Fig. 1. We enrolled a total of 498 healthy term neonates. On screening study subjects, 3 neonates were excluded due to maternal disorders or medications that affect the neonatal coagulation system. Among them, 2 were born to mothers who had congenital antithrombin III deficiency, and 1 was born to a mother who had congenital protein S deficiency. None of the mothers were taking anticoagulants that cross the placenta, such as warfarin and high-dose aspirin, or other medications that inhibit vitamin K (VK) such as anticonvulsants (e.g. phenytoin, barbiturates, carbamazepine), antitubercular drugs (e.g. rifampin, isoniazid), and cephalosporins. In our hospital, an experienced midwife administers all doses of oral VK, and the first dose is given to a neonate after checking that suckling is established by several feeds after birth. During the present study, the compliance to the oral VK was 100 %. The INR values could be measured in 488 of the enrolled neonates. The demographic characteristics and laboratory findings of the study population are shown in Table 1. A comparison of gender, gestational age, birth weight, and hematocrit values during the 4 seasons did not indicate any difference among the 4 groups.

**Table 1:** Demographic data of the study population and outdoor/indoor thermal data byseason

	Spring (*n* = 117)	Summer (*n* = 157)	Autumn (*n* = 96)	Winter (*n* = 118)	*P* value
Male gender, *n* (%)	59 (50)	73 (46)	54 (56)	57 (48)	0.50
Gestational age, wks	39.3 ± 1.1	39.4 ± 1.2	39.5 ± 1.1	39.4 ± 1.2	0.42
Birth weight, g	3078 ± 361	3038 ± 337	3084 ± 370	3100 ± 335	0.49
Hematocrit level, %	51.4 ± 5.6	52.4 ± 5.9	52.0 ± 6.0	52.2 ± 5.6	0.59
INR level	1.07 ± 0.08	1.11 ± 0.10	1.07 ± 0.09	1.06 ± 0.07	< 0.001
Climatic conditions					
Maximum temperature, °C	20.4 ± 3.8	31.2 ± 3.8	22.2 ± 5.8	10.8 ± 2.9	< 0.001
Minimum temperatue, °C	10.6 ± 4.5	23.5 ± 2.6	13.8 ± 5.2	3.1 ± 3.4	< 0.001
Average temperature, °C	15.3 ± 3.8	27.3 ± 3.0	18.0 ± 5.4	7.0 ± 3.0	< 0.001
Relative humidity, %	49.0 ± 20.4	66.0 ± 10.6	56.0 ± 17.2	46.6 ± 15.3	< 0.001
Thermal environment of the newborn nursery					
Room temperature, °C	26.7 ± 0.5	26.0 ± 0.4	26.3 ± 0.5	26.5 ± 0.4	<0.001
Relative humidity, %	31.3 ± 9.1	64.1 ± 6.7	38.9 ± 14.1	25.8 ± 3.3	<0.001

Values are presented as mean ± standard deviation (SD) unless otherwise indicated INR, intenational normalized ratio

### Temperature and humidity variations

**Fig. 2:**
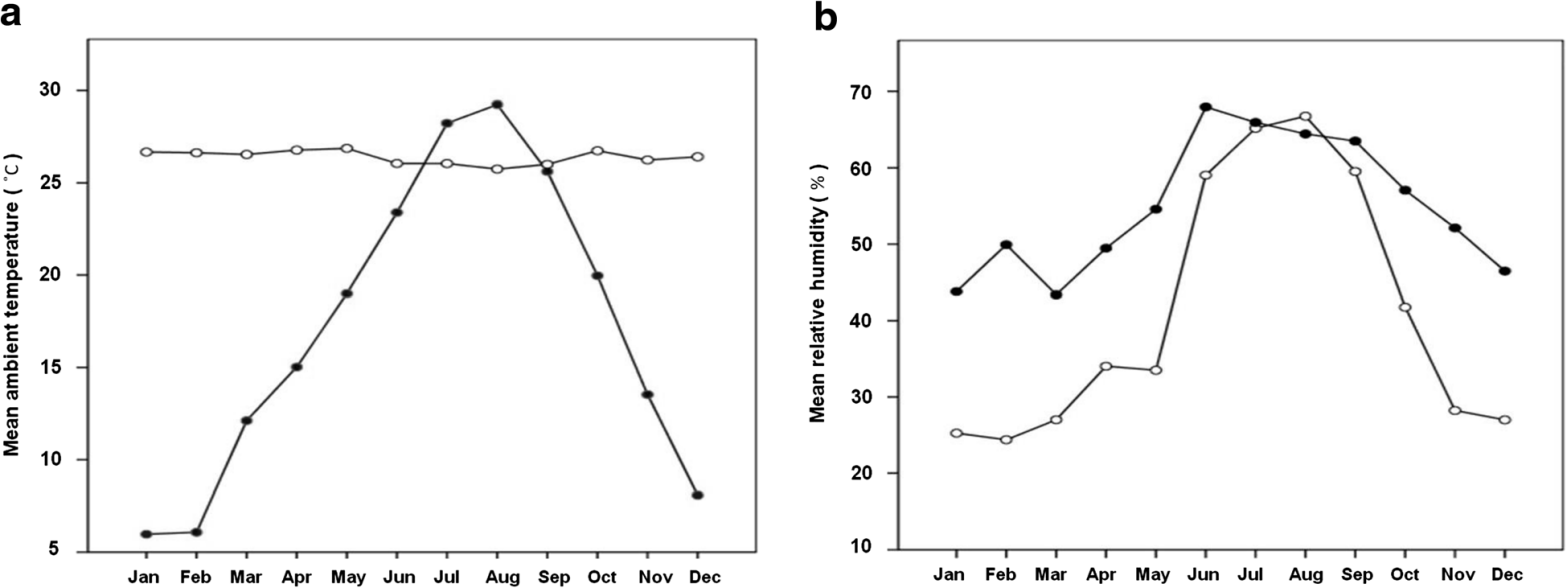
Mean monthly ambient temperature (**a**) and relative humidity (**b**). –●–, Outdoor temperature or relative humidity; −○–, room temperature or relative humidity

The mean monthly outdoor/room temperature and relative humidity are shown in Fig. 2. The outdoor temperature was the lowest in January (6.0 ± 1.9 °C) and peaked in August (29.0 ± 1.5 °C). The outdoor relative humidity peaked in June (65.0 % ± 8.9 %) and was the lowest in January (42.0 % ± 12.1 %). The room temperature was the lowest in August (25.7 ± 0.3 °C) and peaked in May (27.0 ± 0.5 °C). The room relative humidity peaked in July (67.1 % ± 5.9 %) and was the lowest in February (24.3 % ± 2.2 %). A comparison of the climate variables among the 4 seasons indicated that the temperature and relative humidity levels were higher during the summer season. In contrast, with regard to the room thermal environment, the temperature was significantly lower in the summer season (*p* < 0.001), even though the variations were minimal throughout the year. In addition, the relative humidity was significantly higher during the summer season (Table 1).

### Monthly and seasonal variations in the INR values

**Fig. 3:**
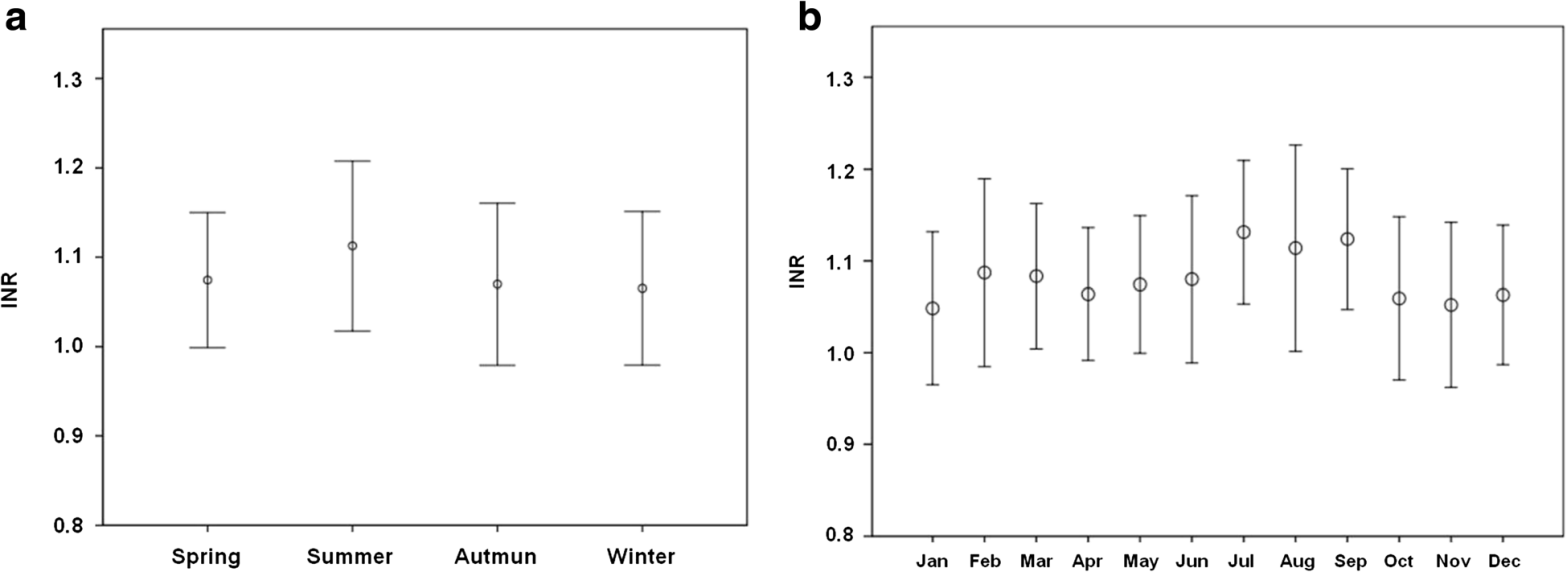
Mean seasonal (**a**) and monthly (**b**) differences in the mean international normalized ratio values, obtained by the CoaguChek XS device

Monthly and seasonal differences in the mean INR values are shown in Fig. 3. The INR values peaked in July (1.13 ± 0.08) and were the lowest in January (1.05 ± 0.08). In fact, the INR exhibited a seasonal pattern on a monthly basis. Over the 4 seasons, the INR values were found to peak in the summer (1.11 ± 0.10), but were the lowest in winter (1.06 ± 0.09); the difference in the INR values among the seasons was statistically significant (*p* < 0.001) (Table 1).

### Correlation factors

**Fig. 4:**
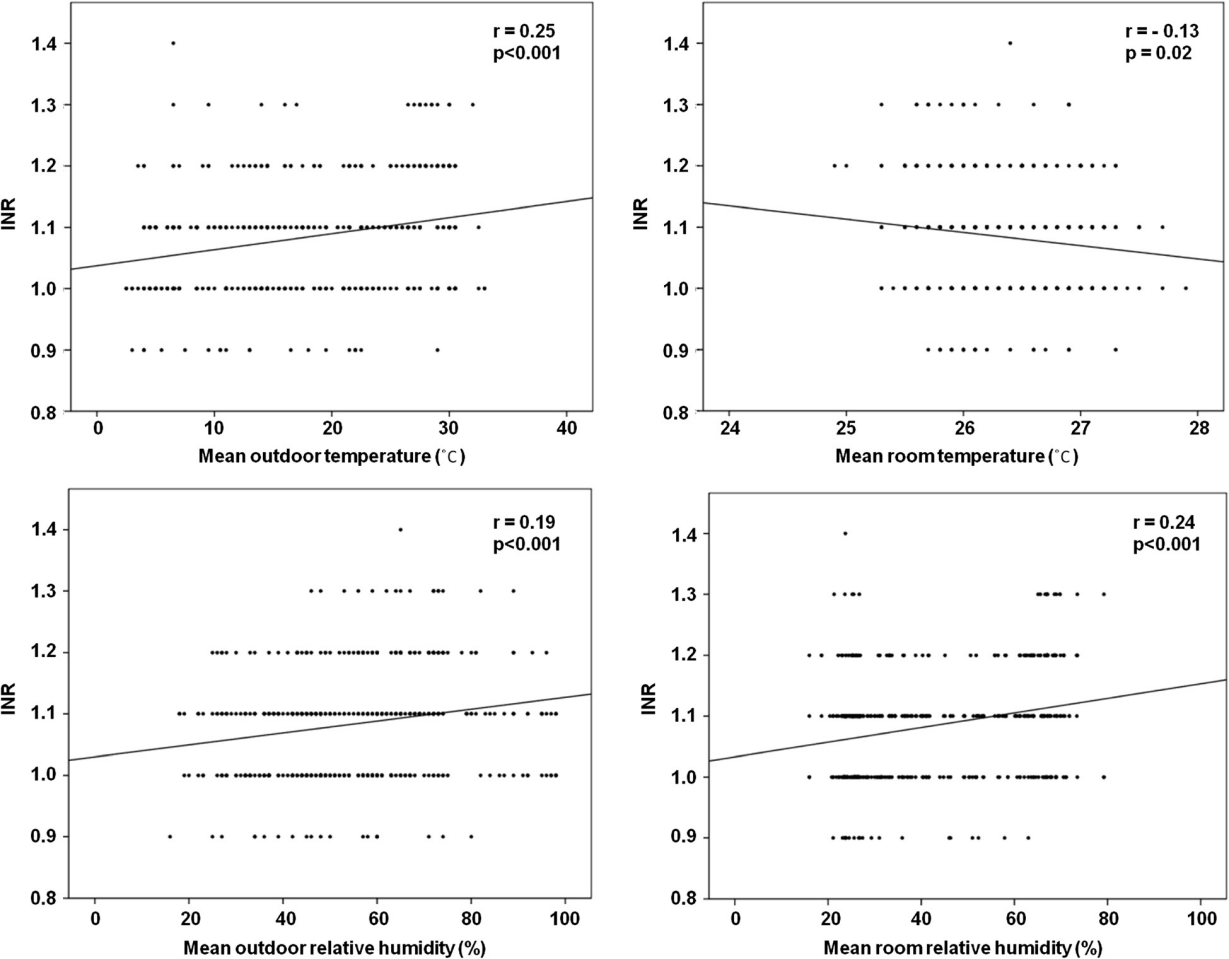
Relationship between the international normalized ratio values obtained by the CoaguChek XS device, and outdoor/room temperature and relative humidity

To assess the influence of environmental variables on INR, we performed simple linear regression analyses with INR as the dependent variable, and outdoor/room temperature and relative humidity as the independent variables. The relationship between INR and each factor is shown in Fig. 4. We observed weakly positive but significant correlations between INR and outdoor temperature (*r* = 0.25, *p* < 0.001), outdoor relative humidity (*r* = 0.19, *p* < 0.001), and room relative humidity (*r* = 0.24, *p* < 0.001). However, there was a weakly negative but significant correlation between INR and room temperature (*r* = −0.13, *p* = 0.02). To determine the reason for the variations in the correlations between INR and outdoor/room temperature, we assessed the relationship between INR and the wind chill temperature (WCT)—the degree of cold perceived by the human body—in the newborn nursery. The WCT represents the combined effects of temperature, humidity, and wind. Using the Missenard formula, the WCT was calculated as follows:

$$ \mathrm{W}\mathrm{C}\mathrm{T}\left({}^{\circ}\mathrm{C}\right)=37\hbox{-} \left(37\hbox{-} t\right)/\left(0.68\hbox{-} 0.0014\times h+1/A\right)-0.29\times t\times \left(1\hbox{-} h/100\right), $$

where *t* is the ambient temperature (in °C) and *h* is the relative humidity (in %). *A* is calculated as: 1.76 + 1.4 × (*v*0.75), where *v* is the wind speed (in m/s) [16]. To evaluate the effect of the WCT on INR, linear regression analyses were performed by using INR as the dependent variable, and WCT (calculated using room temperature and relative humidity data) as the independent variable; for convenience, the wind speed was disregarded in this calculation, as the newborn nursery did not have any open doors or windows, and the neonates were not exposed to wind. Accordingly, we observed a weakly positive but significant correlation between the INR and WCT (*r* = 0.23, *p* < 0.001) in the simple linear regression model.

Thereafter, multiple linear regression analysis was performed to clarify the influence of the independent variables, including WCT, on the INR. We observed that only outdoor temperature significantly influenced the INR values, whereas the other independent variables had no significant correlation with the INR values (Table 2).

**Table 2:** Multiple linear regression model showing association of INRS with predictors

Factor	Regression coefficient	*P* value
Outdoor average temperature	0.25	< 0.001
Outdoor relative humidity	0.09	0.06
Room temperature	−0.22	0.66
Room relative humidity	0.09	0.33
Wind chill temperature in the postnatal ward	0.03	0.48

INRs, international normalized ratios

## Discussion

In the present study, we observed that INR levels were significantly higher during the summer, as compared to those during the winter seasons. This finding could explain why neonatal VKDB more frequently develops during the summer season.

After examining the effect of ambient temperature on seasonal variation, we observed a weakly positive but significant correlation between ambient temperature and the INR values. In the adult population, a prothrombotic state was clearly observed during the winter season [1, 10]. To the best of our knowledge, no study has evaluated the influence of season or temperature on the INR values in children, let alone neonates.

Several blood markers such as fibrinogen exhibit seasonal variations, wherein increases are primarily observed during the cold season; this could be one possible reason for the peak occurrence of cardiovascular events in winter [2, 11, 17]. Previous studies have indicated that a short-term decrease in temperature was associated with increased levels of fibrinogen [12, 13]. Moreover, Stout et al. demonstrated that there was a strong negative relation between both personal and environmental temperature and fibrinogen concentration [11]. In particular, factor VII (FVII) is known to activate the coagulation cascade, and its levels exhibit an increase during winter [2, 18]. Moreover, a positive correlation has been observed between ambient temperature and FVII [10, 14]. Based on these findings, we hypothesized that an ambient temperature change could lead to variations in procoagulant blood markers, such as fibrinogen and FVII, and could consequently influence the INR levels in neonates in the same manner as in adults.

Some of the findings in the present study were interesting. First, the room temperature exhibited a seasonal change, in contrast to the outdoor temperature, even though these variations were minimal. Seasonal changes in room temperature have been found to be significantly lower during the summer than in the other seasons, which could be explained by the action of the air conditioning system. To control the room temperature, cold air is supplied through the ducts of the air conditioner on the roof to the room during the summer season, whereas hot air is supplied in the same manner during the winter season. Second, we observed inversed temperature effects on INR in the newborn nursery, but a positive correlation between the outdoor temperature and the INR values. Other environmental factors such as humidity may explain this difference. The relative humidity exhibited seasonal changes, regardless of whether it was measured outdoors or indoors; this value peaked during the summer season and was the lowest during the winter season. Simple linear regression indicated a significantly positive correlation between outdoor/room relative humidity and INR, although the effect of humidity on the INR values was not observed on multiple linear regression analysis. Furthermore, no study has indicated significant correlations between environmental humidity and the levels of coagulation markers. The WCT, rather than ambient temperature, is used for analysis when considering the influences of a warm or cold environment. In the present study, the WCT recorded in the newborn nursery exhibited a similar seasonality as the outdoor temperature. Although multiple linear regression analysis was conducted by using WCT as an independent variable, no correlation was observed with the INR values, in contrast to outdoor temperature. We have not found any studies reporting on the correlation between the WCT and levels of coagulation markers. Third, in the present study, we observed that the outdoor temperature had an influence on the INR values, even though the subjects received nursing care since birth in a room with a controlled temperature of approximately 26 °C. A previous study in the UK indicated that elderly people residing in centrally heated warden-controlled accommodations exhibited similar seasonal variations in the mortality rates, as compared to those residing in their own homes [19]. With regard to the possible cause, in that study, the authors suggested that the beneficial effects of heating may have been counteracted by the fact that subjects may have spent some time outdoors on cold days. Another study proposed that the central heating may not markedly increase the minimum indoor temperature or affect the core body temperature [11], and low indoor temperatures are reported to be an important factor for increased mortality during winter [20]. Nevertheless, the subjects in the present study were never exposed to the outdoor temperature since birth. Although no data have been provided, the room temperature in the newborn nursery was maintained constant throughout the day. Hence, we believe that the seasonal variations or circannual rhythms in coagulation may be due to factors already present at birth. Animals are programmed by the ambient temperature during the prenatal period to alter their organic responses to changes in ambient temperature. The environmental influences during the prenatal period are thought to induce epigenetic changes in gene regulation [21]. The prenatal development of thermoregulatory mechanisms is also vital for the functioning of the entire organism. Hence, the ambient temperature during pregnancy may have a significant effect on the adaptability of neonates during later life. We speculate that epigenetic programming of the adaptability to seasonal changes in outdoor temperature may affect the coagulation system in early infancy.

In our previous study, we showed that the measurement of INR using a portable coagulometer, CoaguChek XS solved the problem of blood sampling (blood access and blood volume required) for coagulation tests and that it could be a candidate method to screen for VK deficiency. In addition, we established reference intervals of INR in healthy, term neonates [15]. However, the present study demonstrated that it is necessary for temperature to be considered when evaluating INR in early neonates because INR has seasonal variations and is affected by outdoor temperature even if they are cared for in an air-conditioned room. One limitation of the present study was the lack of comparison of the INR values determined by the CoaguChek device with those obtained using a standard laboratory assay or other coagulation parameters. In a previous study, we observed that the INR values determined by the CoaguChek XS were closely correlated with the laboratory INR values (*r* = 0.967), and thus, we clarified the accuracy of the CoaguChek XS device [15]. With regard to the stability of the test strips of the CoaguChek XS device, the manufacturer’s manual clearly states that environmental temperatures ranging between 2 and 30 °C do not significantly affect the test results. The other limitation is the post hoc nature of the analyses. With regard to the data included in the statistical analyses, we used the average outdoor temperature/relative humidity and room temperature/relative humidity recorded at a similar time in the morning on days on which blood samples were obtained. However, the ambient temperature/humidity data were not available at the time points at which blood samples were obtained. Therefore, there is a need for further wide-range prospective studies that include various hematological parameters to clarify the correlation between neonatal hemostasis and ambient temperature or humidity.

## Conclusion

A seasonal variation in the INR values is observed even in neonates, possibly due to variations in the temperature. Interestingly, the outdoor temperature was the most influential factor on the INR values, even though the neonates received nursing care since birth in a newborn nursery that was air-conditioned throughout the day.

## Abbreviations

FVII, factor VII; INR, international normalized ratio; ISI, international sensitivity index; NICU, neonatal intensive care unit; PT, prothrombin time; SD, standard deviation; VKDB, vitamin K deficiency bleeding; WCT, wind chill temperature
